# In Vitro Response of Dental Pulp Stem Cells to Calcium Silicate-Based Cements: A Systematic Review and Meta-Analysis of Preclinical Evidence

**DOI:** 10.7759/cureus.88990

**Published:** 2025-07-29

**Authors:** Ishika Chatterjee, Pratik Agrawal, Yash Sinha, Neelanjana Majee, Sonali Bansal

**Affiliations:** 1 Department of Conservative Dentistry and Endodontics, Kalinga Institute of Dental Sciences, Bhubaneswar, IND; 2 Department of Conservative Dentistry and Endodontics, Kalinga Institute of Dental Sciences, Kalinga Institute of Industrial Technology (KIIT) Deemed to be University, Bhubaneswar, IND; 3 Department of Conservative Dentistry and Endodontics, Postgraduate Institute of Dental Education and Research, Indira Gandhi Institute of Medical Sciences, Patna, IND

**Keywords:** biodentine, calcium silicate cements, cell viability, cytocompatibility, dental pulp stem cells, mta, odontogenic differentiation, regenerative endodontics, systematic review, vital pulp therapy

## Abstract

Regenerative endodontics utilizes stem cell biology and bioactive materials to restore pulp vitality. Human dental pulp stem cells (hDPSCs), with their self-renewal and odontogenic differentiation potential, are central to regenerative endodontics. Hydraulic calcium silicate-based cements (HCSCs), such as mineral trioxide aggregate (MTA) and Biodentine (Septodont, Saint-Maur-des-Fossés, France), are widely used in vital pulp therapies to promote pulp vitality recovery. This research evaluates the response of hDPSCs to HCSCs through a meta-analysis that assesses their cytocompatibility and bioactive effects on hDPSCs in laboratory conditions. The review adhered to Preferred Reporting Items for Systematic Reviews and Meta-Analyses (PRISMA) guidelines and was registered at PROSPERO (CRD42023473456). A complete search of PubMed, Scopus, and Web of Science databases included all scientific articles between 2014 and 2023. Research involving hDPSCs exposed to HCSCs included in vitro investigations that studied both cell survival and proliferation, as well as migration, adhesion, and odontogenic differentiation. The risk assessment for potential bias in the studies used the Cochrane ROB-2 tool. The monitoring process for assessing the effects of pooled cell viability relied on RevMan 5.3 software (The Cochrane Collaboration, London, UK) for the execution of this meta-analysis. Quality synthesis included 16 studies, while the meta-analysis included three studies. The majority of research findings demonstrate that MTA, when combined with Biodentine, particularly at concentrations of 0.2-2 mg/mL, along with accelerated MTA and a calcium-enriched mixture (CEM, BioniqueDent, Tehran, Iran), effectively boosted hDPSC cell viability and migration, while also promoting their odontogenic differentiation. The laboratory tests demonstrated that TheraCal LC (BISCO, Inc., Schaumburg, IL, USA) produced toxic effects, but TheraCal PT exhibited non-toxic properties. Based on meta-analyzed data, the control groups attained a standardized mean difference of -0.26 (95% CI: -0.90 to 0.38), though this difference proved non-significant at p > 0.05. The examination of funnel plots found no indication of important publication bias. The vital pulp treatment success of MTA, Biodentine, accelerated MTA, and CEM as hydraulic calcium silicate-based biomaterials becomes more evident due to their induction of positive hDPSC biological outcomes, such as enhanced cell viability and migration and enhanced odontogenic differentiation potential. In addition, in vivo research, along with clinical trials, must precede material selection optimization for implementing regenerative endodontic treatments.

## Introduction and background

Regenerative dentistry harnesses stem cells and bioactive materials to restore dental tissues. Human dental pulp stem cells (hDPSCs), derived from neural crest cells, exhibit mesenchymal stem cell-like properties, which facilitate dentin structure formation and support periodontal tissue restoration [1]. The exceptional neurodifferentiation and angiogenesis capabilities of hDPSCs make these cells ideal for pulp regeneration [2]. By leveraging the potential of these cells, regenerative treatments can promote tissue repair and renewal, offering promising solutions for dental tissue engineering. Bioceramic materials have revolutionized endodontics with their exceptional biocompatibility and physicochemical properties [3]. Notably, silicate-based materials promote healing, support angiogenesis and tooth development, and reduce inflammation, leading to favorable outcomes.

Hydraulic calcium silicate-based cements (HCSCs) are bioactive endodontic cements that harden in water, producing calcium hydroxide [3]. They are used in various clinical applications, including pulp capping, apexification, and root-end filling, promoting tissue regeneration and hard tissue restoration.

HCSCs are widely used in endodontics due to their bioactive properties. Mineral trioxide aggregate (MTA) was the first commercially available calcium silicate cement, promoting tooth cell differentiation and angiogenesis [4]. However, its prolonged setting time can be reduced with accelerators such as calcium chloride, although some studies have shown adverse effects on dental pulp stem cells [5]. Newer formulations, such as NeoMTA, offer improved properties, including stain resistance and no discoloration effects, making them suitable for vital pulp therapy in both primary and permanent teeth [6,7]. Other materials, such as calcium-enriched mixture (CEM, BioniqueDent, Tehran, Iran) and TheraCal LC (BISCO, Inc., Schaumburg, IL, USA), also demonstrate promising bioactive properties [6,8]. Biodentine (Septodont, Saint-Maur-des-Fossés, France), with its fast-setting time and ability to enhance TGF-β1 production, is effective in regenerative endodontic procedures [9,10]. These hydraulic calcium silicate cements have shown potential in vital pulp therapy and regenerative endodontics, warranting further research on their biological effects and interactions with dental pulp stem cells.

## Review

Methods

A systematic review was conducted following the Preferred Reporting Items for Systematic Reviews and Meta-Analyses (PRISMA) guidelines and registered on PROSPERO (CRD42023473456) [11]. The review searched PubMed, Scopus, and Web of Science for in vitro studies published after 2014, examining the effects of hydraulic calcium silicate cements on hDPSCs.

Criteria for Eligibility

The study design utilized the PICO (patient/problem, intervention, comparison, and outcome) framework. The study subjects consisted of hDPSCs.

Intervention

The intervention involves examining a solution containing hydraulic calcium silicate cements.

Comparison

Tests were conducted on a control group that received no intervention and lacked different amounts of the chosen components.

Results

The application of HCSCs promoted all four essential cellular functions of dental pulp stem cells, including increased proliferation, enhanced migration, and improved cell adhesion, ultimately leading to their odontogenic differentiation.

Study Design

The review included in vitro investigations published between 2014 and 2023.

Review Question

An assessment of hDPSCs (P) occurs when exposed to culture medium containing various HCSCs (I) in comparison to unconditioned culture media (C) regarding their cell survival and proliferation, migration, and odontogenic differentiation (O) through in vitro experiments (S).

Criteria for Inclusion

The review included in vitro studies examining the response of hDPSCs to HCSCs, focusing on cell compatibility, proliferation, migration, adhesion, and odontogenic differentiation. Studies compared HCSCs with and without additives, published in English from 2014 to 2023, with full-text availability.

Criteria for Exclusion

The literature research utilized PubMed, Scopus, and Web of Science as electronic databases to search for papers published between 2014 and 2023, covering the last 10 years. Two different researchers conducted the study selection together with variable extraction and bias risk evaluation. Researchers designed the search methodology based on dental material research literature, which featured their most frequently cited descriptors. The researchers completed the cross-referencing process and conducted a gray literature search using Google Scholar, Greylist, and OpenGrey.

The authors conducted a manual review of the endodontic literature, which included the International Endodontic Journal, Journal of Endodontics, Journal of Endodontology, Saudi Endodontic Journal, Journal of Conservative Dentistry, Australian Endodontic Journal, European Endodontic Journal, British Dental Journal, and Journal of the American Dental Association. The search incorporated relevant keywords and Medical Subject Headings (MeSH) terms through the application of Boolean operators, including "AND" and "OR". The research incorporates the following MeSH: "Tricalcium silicate cement", "Bioceramic", "Dental pulp stem cells", "hDPSC", "Cell viability", "Cell proliferation", "Cell migration", "Biocompatibility", and "Osteogenic differentiation".

Study records from the search were transferred to the Mendeley Desktop version 1.19.4 reference management tool, developed by Elsevier for manual duplicate verification (Elsevier, Amsterdam, The Netherlands). A subsequent check for duplicate records allowed evaluators to review both titles and abstracts based on the previously mentioned criteria. The research adopted a dual-step evaluation procedure to assess articles. At the first stage, two assessors reviewed all articles through their titles and abstracts. Studies that met the established entry requirements were included in the analysis. Each study that passed the established criteria proceeded to full-text screening to determine eligibility for synthesis. The two reviewers conducted a standalone evaluation of the whole articles chosen for assessment in the second phase.

Extraction of Data

The extracted data from the resultant studies formed three distinct categories: research characteristics, methodology, and findings. The study features included several variables, including authors, country, and publication date. The research methodology variables covered the materials with concentrated information about HCSCs, together with described biological examination methods and testing procedures. Each experimental trial yielded significant data points, as reported in the results.

Evaluation Procedure

Two different authors performed the screening and searching procedure. The research was divided into two stages for article selection. The evaluation of all papers began with their titles and abstracts being assessed by two reviewers. The selection process included all articles that met the criteria for acceptance. The same reviewers evaluated and checked the chosen full articles that reached the second stage. The solution of all conflicts required discussion between the parties involved. Two reviewers independently evaluated study references before a third researcher made any necessary final decisions when both initial reviewers did not reach an agreement. The authors' final decision regarding their selections depended upon unanimous agreement between each member. The research authors were contacted via email to obtain any additional information needed.

Evaluation of the Quality of Included Studies

A quality evaluation of the included studies was conducted using the Cochrane Collaboration's Risk of Bias (ROB)-2 tool [12]. The tool assesses seven domains from the ROB-2 tool, which includes random sequence generation (selection bias), allocation concealment (selection bias), personnel and equipment blinding (performance bias), outcome assessment blinding (detection bias), incomplete outcome data (attrition bias), and selective reporting (reporting bias), along with additional biases indicated by their signaling questions in Review Manager (RevMan) 5.3 software (The Cochrane Collaboration, London, UK). The risk analysis for individual research studies received a total evaluation of low, moderate, or high risk based on specific criteria and areas of concern. A study received a low overall risk assessment based on the evaluation of all domains, which were deemed to be of low risk. Research was identified as high risk when it presented any indication of severity in its six domains. The research received a moderate risk assessment when domain uncertainties appeared in one or more sections. No study reached a high risk level.

Quantitative Analysis

The quantitative data analysis computed the standardized mean difference (SMD) along with its 95% confidence interval (CI) to measure continuous outcomes. The study utilized a fixed effects model (Mantel-Haenszel method) except when heterogeneity was detected (p > 0.05 or I-squared < 24%). Then it switched to a random effects model (the Der Simonian-Laird method) [13]. The RevMan version 5.3 software performed all statistical data analysis procedures. The research maintained a significance threshold at P < 0.05.

Evaluation of Heterogeneity

The evaluation of treatment effect variations between different trials utilized Cochran's test, alongside the I² statistic, to determine the proportion of total variability due to heterogeneity above random error. An analysis of statistical heterogeneity revealed significance when the P-value reached less than 0.1. According to the Cochrane handbook, the initial interpretation framework states that heterogeneity ranging from zero to 40% is considered negligible, while a range of 30-60% indicates moderate heterogeneity, a range of 50-90% signifies substantial heterogeneity, and a range of 75-100% reflects considerable heterogeneity [14].

Examination of Publication Bias

The evaluation of publication bias proceeded through assessment of the relative symmetry between individual study estimates and the combined effect size in Begg's funnel plot [15]. A visual depiction of the investigation using the effect size versus standard error method created a funnel plot. The irregular shape of the funnel chart indicates both publication bias and sample size effects as well as genuine relationships between effect measures and study size.

Results

Study Selection

**Figure 1:**
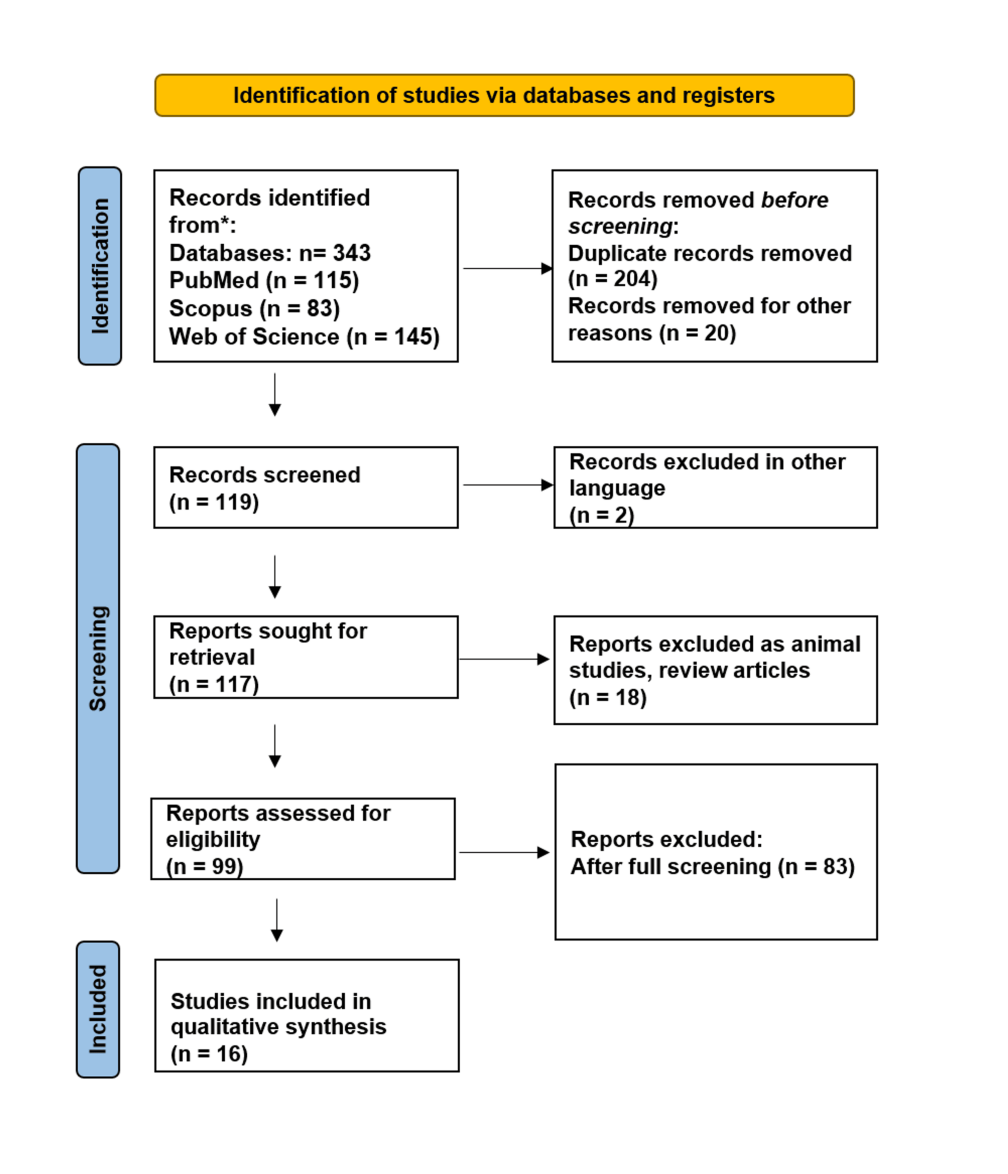
Systematic flowchart representing the study selection process based on the Preferred Reporting Items for Systematic Reviews and Meta-Analyses (PRISMA) flow diagram.

The search yielded 343 records. After duplicate removal and screening, 117 full-text articles were assessed for eligibility. Ultimately, 16 studies were included in the qualitative synthesis, and three studies were included in the meta-analysis. Figure 1 illustrates the flowchart used for identifying studies, along with the inclusion and exclusion processes.

Study Characteristics

The 16 included studies employed a range of assays to evaluate cytocompatibility and cellular responses. Cell viability was commonly assessed using the MTT assay, live/dead fluorescent staining, and flow cytometry. Cell migration was evaluated through wound healing and transwell migration tests, while cell adhesion and morphology were examined using scanning electron microscopy (SEM) and immunofluorescence. Osteogenic differentiation and mineralization were analyzed using alkaline phosphatase (ALP) enzyme activity and alizarin red staining, and qRT-PCR was used to quantify osteogenic and odontogenic markers (Table 1).

**Table 1:** Descriptive study characteristics of the included studies. BA: bio-aggregate; CaCl₂: calcium chloride; CEM: calcium silicate material; HDPSC: human dental pulp stem cells; MTA: mineral trioxide aggregate; NHA: nano-hydroxyapatite; ZnO: zinc oxide eugenol; ALP: alkaline phosphatase

Study	Country	Material Tested	Parameters Evaluated	Key Finding
Youssef et al., 2019 [1]	Saudi Arabia	MTA, Ca(OH)₂, Biodentine, Emdogain	Viability, osteogenic/odontogenic differentiation	MTA, Biodentine, and Emdogain enhanced viability and pulp healing; Emdogain showed potential as an alternative.
Kulan et al., 2016 [5]	Turkey	PMTA with DW, Na₂HPO₄, KY Jelly, CaCl₂	Cell viability (MTS)	Additives improved biocompatibility, offering faster-setting alternatives to conventional MTA.
Tomas-Catala et al., 2017 [7]	Spain	MTA Angelus, MTA Repair HP, NeoMTA Plus	Viability, proliferation, migration	All tested materials showed positive cellular responses, including proliferation and attachment.
Jeanneau et al., 2017 [8]	France	TheraCal LC, Biodentine	DSP expression (Immunofluorescence)	Biodentine significantly increased DSP levels; TheraCal LC was deemed unsuitable for direct pulp capping.
Chang et al., 2014 [16]	Korea	Bioaggregate, Micromega MTA, ProRoot MTA, IRM	Cell viability, Odontogenic differentiation	BA and MMTA demonstrated similar biocompatibility and regenerative potential to PMTA, which is superior to IRM.
Jaberiansari et al., 2014 [17]	Iran	PMTA, AMTA, RMTA, CEM, NMTA	Cell viability	All MTA variants and CEM enhanced DPSC proliferation, but NMTA showed cytotoxic effects.
Luo et al., 2014 [18]	China	Biodentine (0.02–20 mg/mL)	Proliferation, migration, adhesion	Lower concentrations of Biodentine enhanced DPSC functions; higher doses decreased viability.
Bortoluzzi et al., 2015 [19]	Brazil	Biodentine, TheraCal LC, MTA Angelus	Viability, ALP activity, gene expression	Biodentine promoted the highest viability and gene expression; TheraCal LC was cytotoxic.
Niu et al., 2015 [20]	China	Quick-Set 2, White MTA	Viability, proliferation	Quick-Set 2 was less cytotoxic than WMTA, likely due to its lower pH.
Mohamed et al., 2017 [21]	Egypt	NHA, MTA, CEM	Viability, ALP activity	MTA and CEM were favorable for DPSCs; NHA exhibited cytotoxicity.
Kulan et al., 2018 [22]	Turkey	PMTA with DW, CaCl₂, Na₂HPO₄	Viability, ALP activity, RT-qPCR	Accelerated MTA improved viability and differentiation compared to unmodified PMTA.
Pedano et al., 2018 [23]	Belgium	PPL-CSC, Nex-CEM MTA, Biodentine, ZnOE	Proliferation, migration, differentiation	All CSCs tested were non-cytotoxic and supported DPSC activity; PPL-CSC was promising.
Tomas-Catala et al., 2018 [24]	Spain	NeoMTA Plus, MTA Repair HP, Biodentine	Viability, migration, cytotoxicity	All materials showed acceptable cytocompatibility; Biodentine had the highest proliferation rates.
Omidi et al., 2019 [25]	Iran	TheraCal LC, AMTA, CEM, Biodentine	Viability, migration, cytokine expression	CEM and Biodentine yielded the highest viability and TGF-β1 expression; TheraCal LC was cytotoxic.
Sun et al., 2019 [26]	China	Biodentine, iRoot FS	Viability, migration, differentiation	Both materials were bioactive; iRoot FS demonstrated better migration and proliferation.
Sanz et al., 2021 [27]	Spain	TheraCal PT, TheraCal LC, Biodentine	Viability, migration, adhesion, marker expression	TheraCal PT showed improved bioactivity over TheraCal LC, with comparable results to Biodentine.

Study Results

The study findings indicated that MTA, Biodentine, and rapid-set MTA with setting-time reducers promoted better cell viability compared to the negative control. Emdogain showed promising results as an alternative to MTA and Biodentine for dental pulp regeneration. In contrast, TheraCal LC exhibited toxic effects on DPSCs, while its updated version, TheraCal PT, demonstrated improved cell compatibility. Biodentine showed higher cell viability than MTA in some settings. Accelerated MTA with CaCl₂ and Na₂HPO₄ was compatible with DPSCs, and Biodentine's effect on cell proliferation varied with different concentrations, and this enhancement disappeared at concentrations of 20 mg/mL, indicating that the amount of capping substance affects cellular viability [18].

Biodentine, MTA, and other materials, such as NeoMTA Plus and MTA Repair HP, promoted cell migration in hDPSCs, with Biodentine showing the highest rate [24]. iRoot Fast Set also enhanced cell proliferation and migration [26]. TheraCal LC negatively impacted cell migration, whereas TheraCal PT showed improved results [27]. Bioactivity tests revealed superior mineralization potential in MTA, Biodentine, and accelerated-set MTA [19,26]. Biodentine and iRoot Fast Set demonstrated promising ALP expression and DSPP expression, indicating potential for dental tissue engineering [19,26].

**Figure 2:**
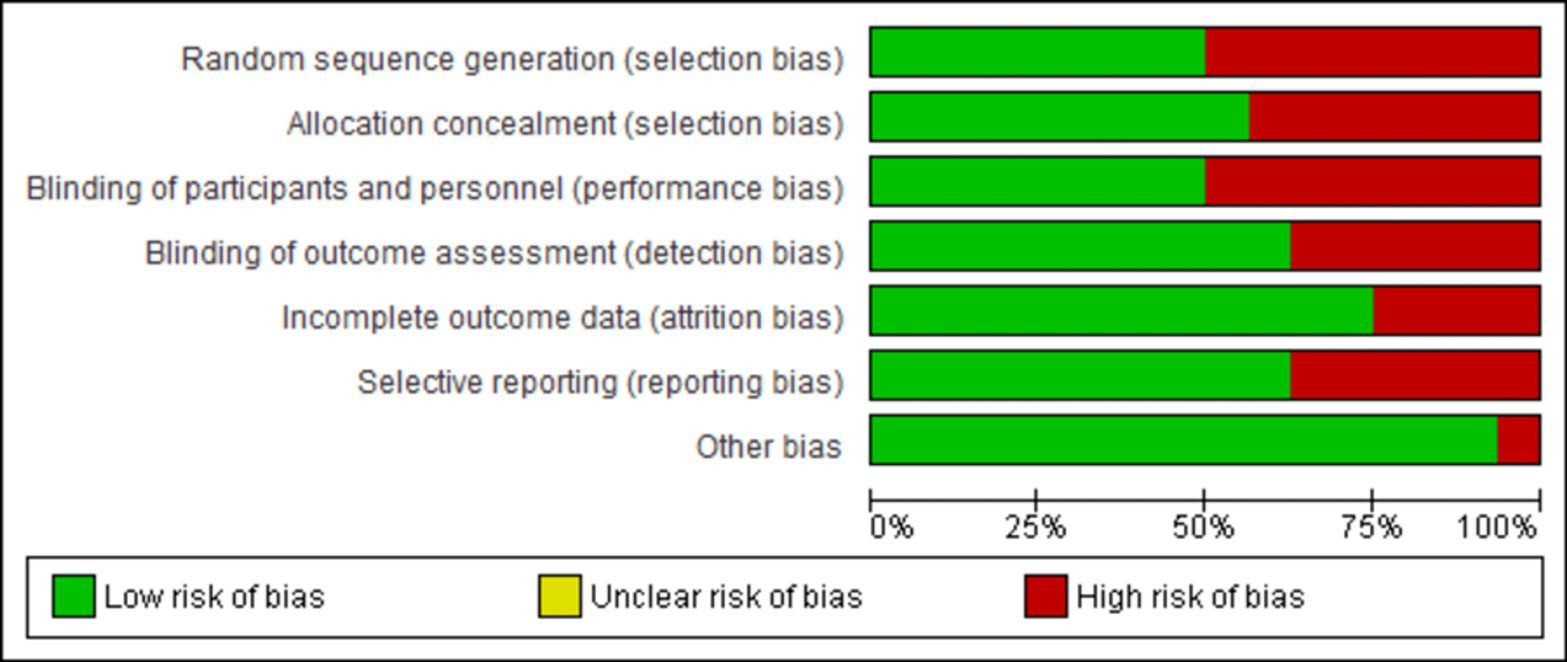
Risk of bias graph showing the review authors' judgements about each risk of bias item presented as percentages across all included studies. The Cochrane Risk of Bias Tool (RoB 2.0) is used in this study.

**Figure 3:**
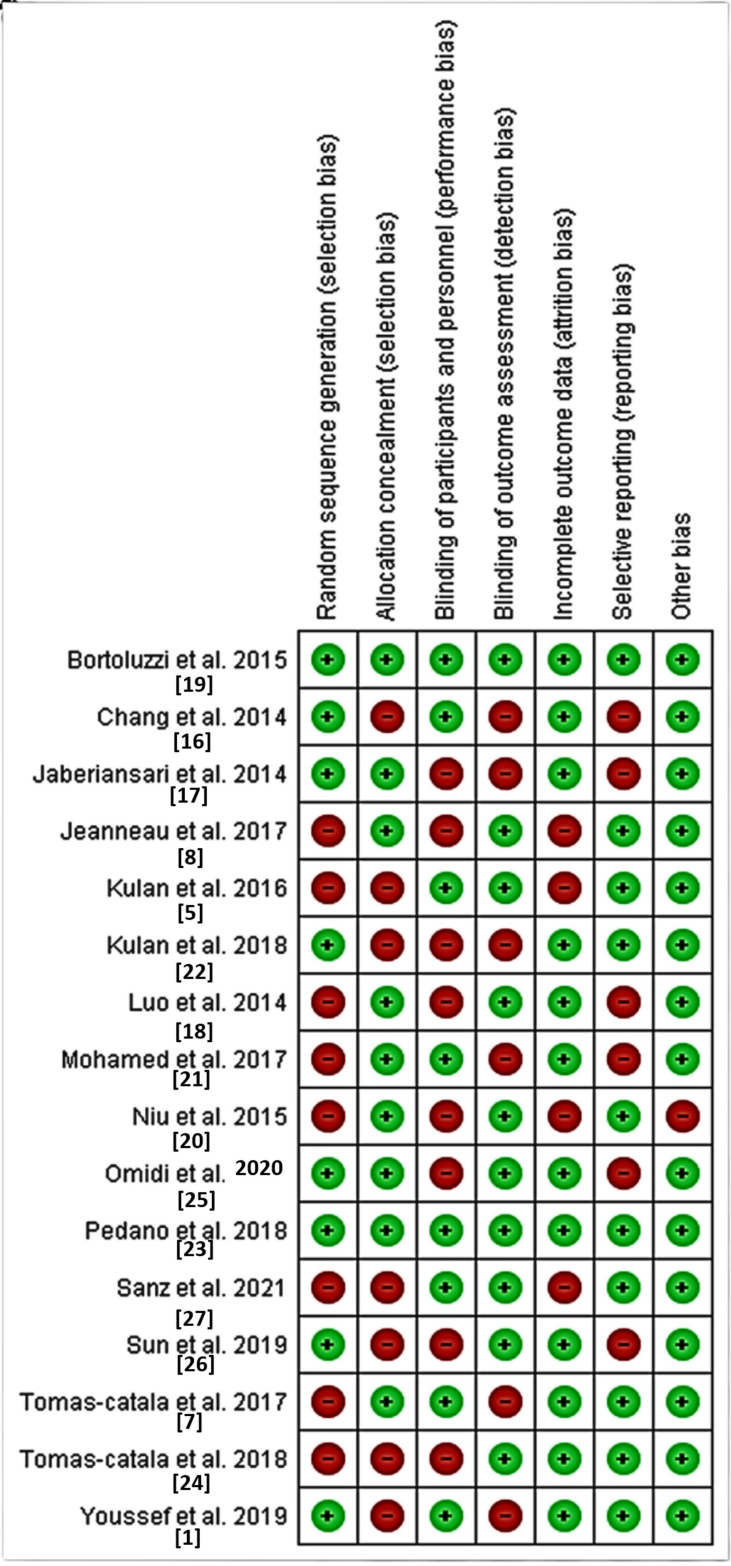
Risk of bias summary showing the review authors' judgements about each risk of bias item for each included study.

All researchers assessed the methodological quality of the studies under evaluation. The methodological consistency across all analyzed studies reached a very high standard. The risk of bias level was identified as moderate to high in all examined studies across all important assessment domains. The highest bias risks emerged from performance bias through participant and personnel blinding and selection bias in random sequence generation. The studies done by two authors demonstrated the lowest possible risk of bias among all researched studies [19,23]. The validated studies focused on rating domains for attrition bias, reporting bias, detection bias, and other biases, which presented the least risk to the study outcomes. The Cochrane Risk of Bias (RoB)-2 method (RoB 2.0) revealed that the risk of bias in the included studies is evident (Figures 2, 3).

**Figure 4:**
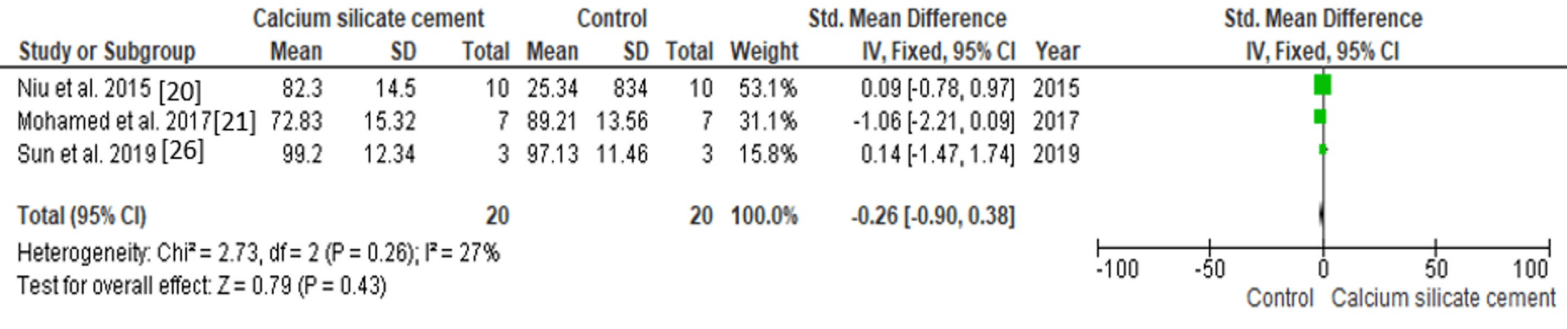
Comparison between calcium silicate cement and the control group for greater mean % viability of dental pulp stem cells.

The SMD serves as a summary statistic for continuous outcomes, assessing the mean percentage viability of dental pulp stem cells in relation to calcium silicate cements vs. controls, as illustrated in Figure 4. Data were assessed from three studies involving a total of 40 specimens, with 20 specimens subjected to calcium silicate cements and 20 specimens serving as controls, to determine the superior efficacy of the two modalities based on the mean percentage viability of dental pulp stem cells. Figure 4 illustrates that the SMD is -0.26 (-0.90 to 0.38), indicating that the pooled estimates favor the control group. This indicates that the mean percentage vitality of dental pulp stem cells is, on average, 0.26 times lower in the control group, and this difference is not statistically significant (p>0.05). Among the included studies, the highest weightage was given by Niu et al. (2015) [20], while the lowest weightage of the study was given by Mohamed et al. (2017) [21].

**Figure 5:**
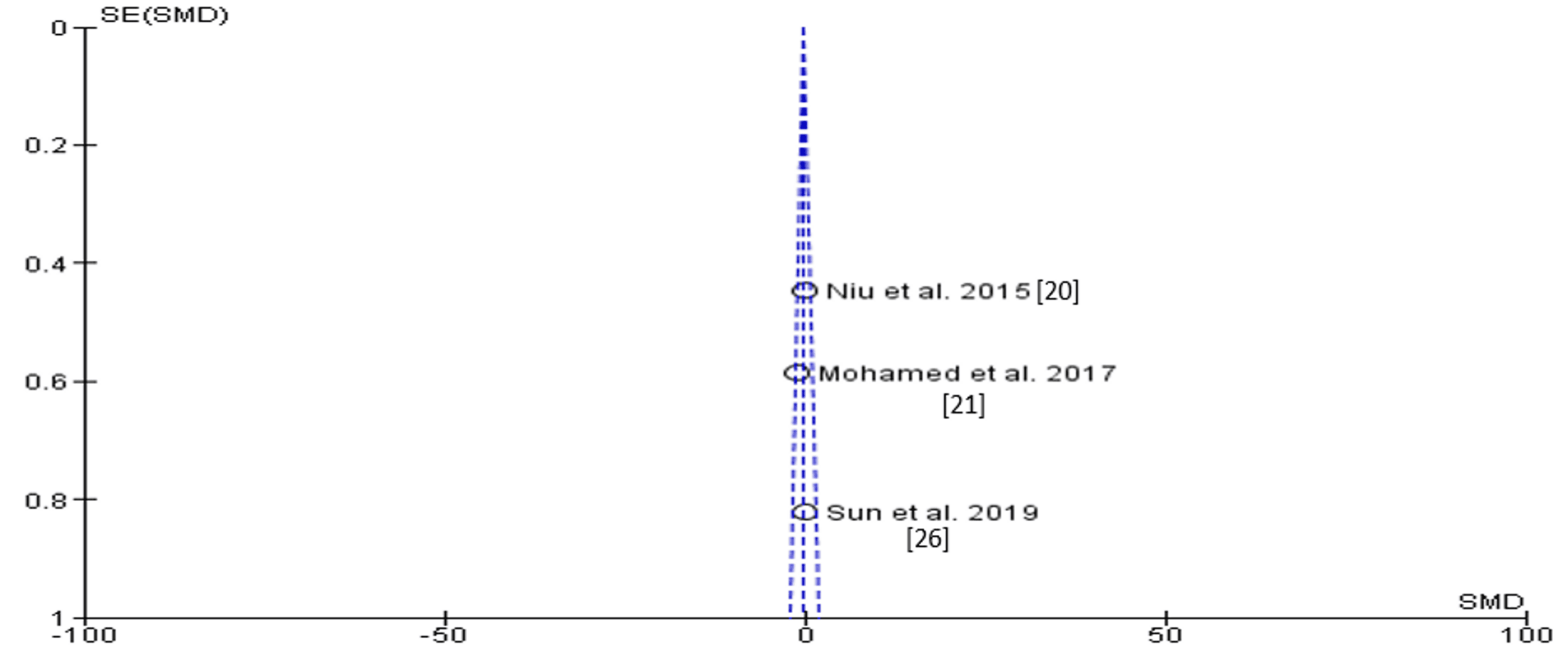
Funnel plot showing an asymmetric distribution, with the absence of systematic heterogeneity of individual studies compared to the standard error, indicating the absence of publication bias in the meta-analysis.

The funnel plot did not show significant asymmetry, indicating the absence of publication bias, as shown in Figure 5.

Discussion

This review assessed the effects of tricalcium silicate-based cements on hDPSC vitality using available research findings, involving cytocompatibility and bioactivity tests with control groups for comparison. hDPSCs treated with Biodentine, MTA, and other materials showed improved viability and migration compared to controls. These materials also enhanced mineralization and ALP activity. Bioaggregate and MicroMega MTA demonstrated biocompatibility and osteogenic responses similar to ProRoot MTA [16].

Enamel matrix derivative, Emdogain, exhibits the lowest toxicity to hDPSCs in comparison to MTA, Ca(OH)₂, and Biodentine. The study demonstrated that Emdogain offers a viable alternative to MTA and Biodentine for promoting pulp healing following dental traumatic events [17].

Research was conducted to investigate the dose-dependent effects of Biodentine on the cytocompatibility, migration, and adhesion of hDPSCs. Optimal cell growth was observed at concentrations of 0.2 mg/mL and 2 mg/mL, whereas 20 mg/mL exhibited cytotoxicity. A concentration of 0.02 mg/mL yielded results comparable to the control group [18].

Studies have shown that tricalcium silicate-based cements, such as Biodentine, NeoMTA Plus, and MTA Repair, facilitate hDPSC migration, with Biodentine exhibiting superior efficacy [24]. Similarly, CEM and Biodentine have been found to significantly enhance hDPSC migration, outperforming TheraCal and MTA [25]. CEM has also demonstrated a notable proliferative effect on DPSCs. The dosage of these silicate-based cements affects their ability to facilitate cell migration.

The osteo/odontogenic differentiation potential of hDPSCs in response to various HCSCs has been investigated through RT-qPCR analysis of marker gene expression. Studies have consistently shown that hDPSCs respond to HCSCs by upregulating multiple osteo/odontogenic markers, including DSPP, DMP-1, ALP, OCN, ON, OPN, BSP, and Runx-2. Notably, MTA and Biodentine have been found to promote robust expression of these markers, with Biodentine exhibiting the highest level of expression. However, variations in ALP expression have been observed, with some studies reporting decreased ALP expression in response to Biodentine and iRoot Fast Set or Biodentine and TheraCal PT [26,27]. Other HCSCs, such as accelerated MTA, iRoot Fast Set, TheraCal PT, and Emdogain, have also been shown to modulate the expression of specific osteo/odontogenic marker genes, highlighting the complexity of hDPSC responses to these materials.

The cytocompatibility and bioactivity of tricalcium silicate-based biomaterials have been extensively studied in the context of hDPSC interactions. Notably, TheraCal LC, a resin-modified calcium silicate material, has been shown to produce adverse effects on hDPSCs, compromising their cytocompatibility and bioactivity [27]. Furthermore, TheraCal LC treatment diminished ALP enzyme activity, indicating impaired osteogenic maturation of hDPSCs compared to Biodentine and MTA.

This systematic review and meta-analysis assessing hDPSC viability in response to HCSCs revealed a non-significant difference in mean percentage vitality between calcium silicate cement groups and control conditions, with the control group exhibiting a 0.26 times lower viability rate (p>0.05). The absence of publication bias in the meta-analysis lends credibility to the findings. Conversely, multiple studies have demonstrated that hDPSCs exposed to various calcium silicate-based products exhibit enhanced survival, growth rates, migration abilities, and mineralization nodule formation, supporting their potential utility in endodontic treatments.

Among the examined HCSCs, MTA, Biodentine (at 0.2 mg/mL and 2 mg/mL concentrations), accelerated MTA, and CEM displayed pronounced cytocompatibility and osteogenic differentiation effects on DPSCs. The development of TheraCal PT has shown an improved hDPSC response to treatment and mineralization compared to its predecessor, TheraCal LC, while sharing similar biological characteristics with Biodentine.

While the reviewed research provides valuable insights into cell reactions to bioactive substances, it is essential to acknowledge the limitations of these investigations, primarily their in vitro nature and the variability in experimental methods and sample sizes. Future studies should focus on multi-stage randomized controlled trials and in vitro examinations to evaluate the sustained effects and clinical outcomes associated with the use of these biomaterials in practice, ultimately determining the most effective techniques for endodontic applications.

## Conclusions

This systematic review and meta-analysis evaluated the biological response of hDPSCs to HCSCs, emphasizing their cytocompatibility, proliferative capacity, migration potential, and odontogenic differentiation. Among the tested materials, Biodentine, MTA, accelerated MTA, and CEM consistently demonstrated favorable bioactivity profiles, supporting their application in vital pulp therapy and regenerative endodontic procedures. While the quantitative meta-analysis revealed no statistically significant difference in cell viability compared to controls, the qualitative synthesis highlights the dose-dependent and material-specific effects of HCSCs on hDPSC behavior. However, the in vitro nature of the included studies and limited meta-analytical data underscore the need for standardized experimental protocols and robust in vivo and clinical investigations to validate translational relevance and optimize biomaterial selection for regenerative endodontic applications.
